# Dyskinesia as a unique presentation of subarachnoid hemorrhage: a case report

**DOI:** 10.1186/s12245-023-00476-2

**Published:** 2023-01-05

**Authors:** Aleq Jaffery, Herman Morchel, Jessica Poon

**Affiliations:** 1 Department of Emergency Medicine, Hackensack University Medical Center, 30 Prospect Ave, Hackensack, NJ 07601 USA; 2 Hackensack Meridian School of Medicine, 340 Kingsland St, Nutley, NJ 07110 USA

**Keywords:** Diagnostic error, Heuristics, Subarachnoid hemorrhage, Metacognition, Case report

## Abstract

**Background:**

Subarachnoid hemorrhage (SAH) is a diagnosis that emergency physicians must have a high index of suspicion for. Many common chief complaints such as headache, nausea, altered mental status, and even syncope may alert clinicians to the possibility of a SAH.

**Case presentation:**

The authors present an unusual case of SAH in a patient presenting with acute dyskinesia and altered mental status, which has seldom been documented as the presenting feature of SAH, as well as the diagnostic pitfalls encountered in assessing this patient.

**Conclusion:**

Emergency physicians should maintain a high index of suspicion for dangerous pathology in cases without a clear etiology; they should also utilize metacognition to assess their own biases and thought patterns so as to avoid missing critical diagnoses.

## Background

Although the worldwide incidence of subarachnoid hemorrhage (SAH) is low at 9 per 100,000 person years, and has been steadily decreasing [1], missed diagnoses can be catastrophic. Up to 5.4% of cases are missed in the emergency department [2]. Indeed, even when properly diagnosed and managed, survivors can have persistent neurologic deficits [3], making this an essential do-not-miss diagnosis. SAH is divided into two categories which differ widely in terms of morbidity, mortality, and management: non-traumatic and traumatic SAH. The vast majority of non-traumatic SAHs are caused by ruptured intracranial aneurysms [4], and the classic presentation taught is that of a thunderclap headache, the “worst headache I’ve ever had,” which is maximal in onset and may be associated with a decreased level of consciousness. It is important to note that less than a quarter of patients who present with a classic thunderclap headache are eventually diagnosed with SAH [4]. Common presenting symptoms include headache, nausea/vomiting, syncope, and neck pain [5]. Clinicians should obtain a thorough history of present illness which includes patient specific risk factors such as family history of SAH or intracranial hemorrhage, drug use with methamphetamines and cocaine being particularly dangerous [6] tobacco use and hypertension history. Physical exam should include most aspects of the neurologic examination including serial Glasgow Coma Scale evaluations if possible, gait, and coordination. Isolated pupillary palsies have been associated with SAH and other intracranial pathology [7], so cranial nerve testing is imperative. Indeed, an isolated neurological exam finding may be the only indication of serious pathology, especially when the patient is unable to provide a good history. A focal neurologic deficit can be identified in 1 of every 10 patients with the aneurysmal variety of SAH, and has also been independently associated with poor outcomes [8]. In this case, we present a patient whose only complaints were altered mental status and acute dyskinesia. Careful neurologic examination, and a willingness to check cognitive biases, were what allowed us to accurately diagnose SAH in this undifferentiated patient.

## Case presentation

A middle-aged female was brought to the emergency department by emergency medical services (EMS) with a chief complaint of altered mental status. Per EMS report, this patient was out with her friends when she went to a restroom and did not emerge for a period of time. When her friends went to check on her, they found her on the ground, covered in vomitus, screaming nonsensical syllables, and smacking her lips. EMS found the patient soiled from head to toe in vomit, intermittently making loud incoherent noises, and with florid dyskinesia of the face consisting of lip smacking, tongue protrusion, and grimacing. Transport to the emergency department was uneventful; however, she was unable to meaningfully interact and there was no access to collateral information or patient identification. Physical exam in the department was significant for dyskinesia and vocal tics, but notably on the bilateral lower extremities there was no writhing movement and the patient did not withdraw to deep painful stimuli. Most other aspects of the exam were limited due to the patient’s inability to follow commands or express herself. She did have reactive pupils and appeared to have no gaze palsy. Initial differential diagnosis included drug-induced dystonia. This case occurred in a state which had recently legalized marijuana and tetrahydrocannabinol products, so there was an anecdotal increase in the treatment team’s practice of dystonic reactions, vocal tics, and even florid psychosis from overdose of cannabinoid products. Paresis of the extremities, however, was not a routine feature seen in the team’s practice environment, prompting them to obtain computed tomography (CT) to rule out intracranial pathology.

**Fig. 1:**
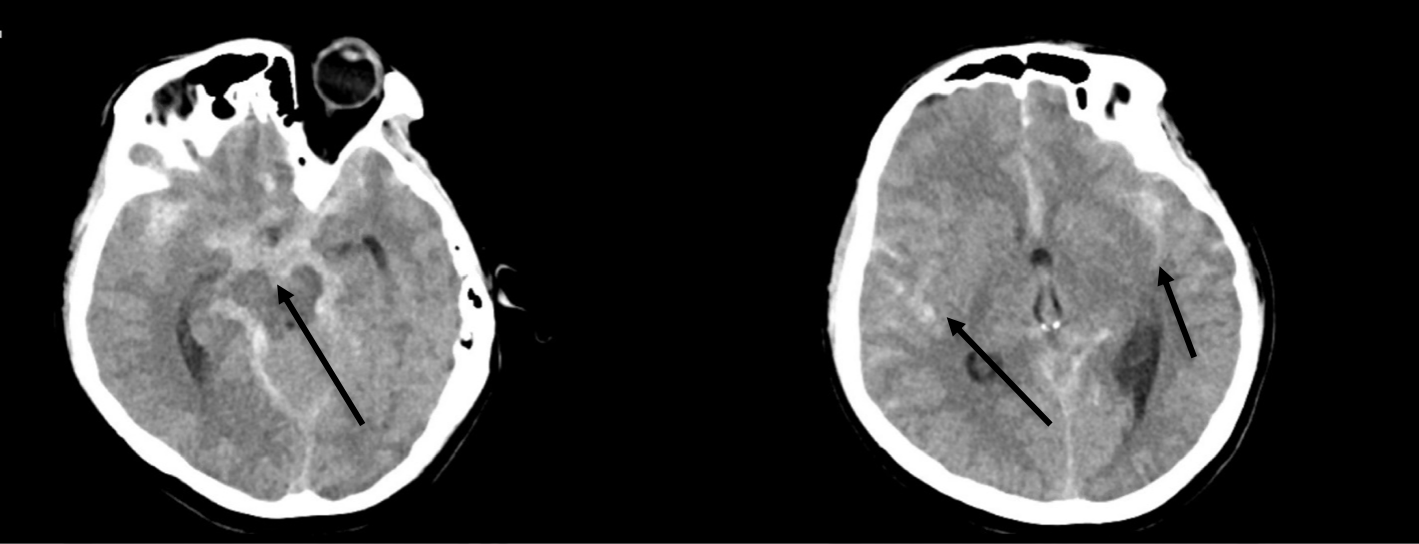
Computed tomography of the head showing large subarachnoid hemorrhage and blood in the fissures (arrows)

CT showed a significant subarachnoid hemorrhage with bleeding involving the basal cisterns, bilateral sylvian fissures, and left greater than right cerebral convexities (Fig. 1). Subsequent CT angiography revealed aneurysms of the anterior communicating artery, right internal carotid aneurysms, and singular right middle cerebral artery aneurysm at the bifurcation. The patient was intubated for airway protection, started on titratable blood pressure medication, and neurosurgery was consulted emergently for coiling of the aneurysms. The patient went directly to procedure for coiling of a 5.5-mm aneurysm of the anterior communicating artery and placement of a right sided external ventricular drainage device (EVD). She then spent a month in the surgical intensive care unit where her course was complicated by hydrocephalus necessitating placement of an additional left-sided EVD, dysphagia, and persistent neurologic deficits. She was discharged after 1 month to a rehabilitation facility neurologically intact except for minimal residual weakness of the distal lower extremities.

## Discussion

SAH is an essential emergent diagnosis with high morbidity and mortality requiring prompt recognition. Diagnosis is made by CT and, if non-diagnostic and/or more than 6 h have passed, lumbar puncture to evaluate for xanthochromia if there is a high enough clinical suspicion [3]. Once a diagnosis of SAH is made, elucidating the cause will guide management. Traumatic SAHs can generally be monitored with or without seizure prophylaxis, but in a non-traumatic SAH, evaluation should include CT angiography to evaluate for intracranial aneurysms. The cornerstone of management for all SAHs is blood pressure control, which has shown to prevent re-bleeding and ischemic damage and improve mortality [9]. Titratable agents such as nicardipine are recommended initially as patients require a fine level of control to maintain cerebral perfusion pressure [3]. Early consultation with neurosurgical services for definitive aneurysm management should occur in the emergency department. As always, high-quality supportive care should be initiated, which includes avoiding hypoxia and hyperglycemia, reversal of and/or withholding anticoagulants, and airway management if mental status continues to deteriorate. If patients are able to tolerate oral medications, nimodipine has been shown to improve neurologic outcomes although it does not improve vasospasm as previously thought [10].

In atypical presentations such as this one, it is important for clinicians to take a step back and consider the case as a whole as well as their own cognitive processes. This case illustrates the availability heuristic, where a familiar presentation limited the diagnosis of choice whatever was available, in this case drug-induced dopaminergic symptoms. This heuristic and other diagnostic errors like it are more morbid and mortal than other healthcare-associated errors [11], particularly when made in the emergency department [12]. It behooves us to put a pause on our diagnostic machinery and ask ourselves “what else might this be?” Studies on preventing diagnostic errors in medicine routinely identify a thorough and comprehensive differential diagnosis list, with multiple pathologies considered, as a protective factor [13].

Limitations of this case are, of course, the solitary patient and inability to draw conclusions regarding the epidemiology of SAH presentation. However, as this case illustrates, the diagnosis of SAH is not nearly as cut-and-dry as a maximal onset thunderclap headache. Clinicians should be careful to judge the history of present illness, risk factors, and perform thorough, detailed, and repeated physical exams in an unclear picture. In this case, the availability heuristic interfered with the correct diagnosis as the patient’s presentation was similar to several recent cases involving cannabinoid intoxication. It is important to note that organic pathology and intoxication not mutually exclusive. In fact, alcohol intoxication has been independently associated with mortality in trauma patients [14]. Emergency providers should not assume that intoxication is the only explanation of a patient’s symptoms; indeed, this woman may well have been intoxicated on cannabis in addition to having a subarachnoid hemorrhage. In cases with atypical presentations or unclear etiologies for symptoms, it is essential to seek information and diagnostics, particularly prior to premature closure, to either push one towards or away from a working diagnosis. This case offers an unusual presentation of a highly morbid pathology but this applies to all cases when a provider is unsure of a diagnosis. It is critical to have a broad differential and utilize metacognition to identify potential diagnostic errors and sources of bias. Asking the simple question “what else might this be?” is an important part of identifying unusual presentations of disease, particularly those with catastrophic courses.

## Data Availability

N/A

## Abbreviations

SAH: Subarachnoid hemorrhage
EMS: Emergency medical services
CT: Computed tomography
EVD: Extra-ventricular drainage
